# The randomized study of enteral nutrition with rapid versus conventional administration in acute stroke patients; the protocol of rapid EN trial

**DOI:** 10.3389/fneur.2024.1393345

**Published:** 2024-06-03

**Authors:** Kentaro Suzuki, Hidetaka Onodera, Rie Sugiyama, Seiji Okubo, Naoto Kimura, Shogo Kaku, Rieko Seki, Satoshi Fujita, Koichi Nomura, Taiki Takagiwa, Izumi Katafuchi, Homare Nakamura, Takuya Kanamaru, Momoyo Oda, Shohei Kimura, Shota Sonoda, Hiroto Kakita, Toshiaki Otsuka, Kazumi Kimura

**Affiliations:** ^1^Department of Neurology, Nippon Medical School, Tokyo, Japan; ^2^Department of Neurosurgery, St. Marianna University School Toyoko Hospital, Kawasaki, Kanagawa, Japan; ^3^Emergency and Critical Care Center, Nippon Medical School, Tokyo, Japan; ^4^Department of Cerebrovascular Medicine, NTT Medical Center Tokyo, Tokyo, Japan; ^5^Department of Neurosurgery, Iwate Prefectural Central Hospital, Morioka, Iwate, Japan; ^6^Department of Neurosurgery, Neurosurgical East Yokohama Hospital, Yokohama, Kanagawa, Japan; ^7^Department of Neurosurgery, Shimizu Hospital, Kyoto, Japan; ^8^Department of Neurosurgery, Toho University Ohashi Medical Center, Tokyo, Japan; ^9^Department of Neurology, Shioda Hospital, Chiba, Japan; ^10^Stroke Care Unit, Nippon Medical School, Tokyo, Japan; ^11^Department of Neurosurgery, St. Marianna University Yokohama Seibu Hospital, Yokohama, Kanagawa, Japan; ^12^Department of Neurology, Iwate Prefectural Central Hospital, Morioka, Iwate, Japan; ^13^Department of Public Health, Nippon Medical School, Tokyo, Japan

**Keywords:** stroke, enteral nutrition, rapid administration, speed, enteral feeding

## Abstract

**Rationale:**

Enteral nutrition is beneficial for stroke patients with oral intake difficulties. However, it is time consuming and may interfere with routine medical care. Therefore, there is a clinical benefit if enteral nutrition can be safely administered in a short time. Although our retrospective study showed the safety of rapid administration, it remains unclear whether rapid administration of enteral nutrition is as safe as conventional administration.

**Aim:**

The randomized study of Enteral Nutrition with Rapid versus conventional administration in acute stroke patients (Rapid EN trial) aims to clarify the safety of rapid feeding of enteral nutrition compared with conventional feeding.

**Methods and design:**

This is an investigator-initiated, multicenter, prospective, randomized, open-label, blinded end-point clinical trial. Eligible criteria include acute stroke patients who have difficulty with oral intake defined as severe altered consciousness (Japan Coma Scale 10–300) or modified water swallowing test <4. The target enrollment is 700 patients, with 350 patients receiving rapid enteral nutrition at a rate of 100 mL in 5 min (Rapid EN group) and 350 patients receiving conventional enteral nutrition at a rate of 100 mL in 30 min (Conventional EN group).

**Study outcome:**

The primary outcome is the incidence of one or more complications of vomiting or diarrhea or pneumonia within 7 days would be non-inferior in the rapid EN group compared to the conventional EN group. Secondary outcomes were total time spent on enteral nutrition within 7 days from enteral nutrition, the incidence of vomiting, diarrhea and pneumonia within 3 or 7 days, and the rate of favorable clinical outcome.

**Discussion:**

Since no previous reports have focused on the speed of administration, we felt it was necessary to prove the safety of rapid administration. If this study shows positive results, it will not only benefit patients, but also reduce the burden of medical care. We believe this study is novel and will be useful in clinical practice.

**Clinical trial registration:**

https://rctportal.niph.go.jp/s/detail/um?trial_id=UMIN000046610 Identifier UMIN000046610.

## 1 Introduction

Malnutrition was observed in 6.1–62% of stroke patients. Nutritional assessments of older patients in rehabilitation and subacute hospitals after acute treatment revealed that 50.5% were malnourished (1), and these patients with malnutrition at the time of transfer had a lower rate of returning home (2, 3). Nutritional interventions in the acute phase after a stroke may prevent the increased infection risk associated with malnutrition and avoid rehabilitation delays due to weight loss and muscle weakness. Studies in the intensive care unit and on trauma patients during the acute phase have reported that early initiation of enteral nutrition (EN) reduces the incidence of infection and mortality (4, 5).

Early nutrition initiation effectively improves malnutrition, and EN is beneficial for patients with difficulties in oral intake. According to the most recent guidelines, in cases where severe dysphagia is expected to last >7 days, enteral tube feeding should be initiated within the first 72 h after stroke onset (6, 7). However, early initiation of EN is associated with risks, such as vomiting, diarrhea, and aspiration pneumonia (8). The recommended slow and diluted administration method to manage risk is time-consuming and may interfere with routine medical care. In addition, the effectiveness of slow and diluted administration is not clearly evident. The acute phase of stroke management requires time to spend on intravenous drip infusion, frequent imaging studies, and rehabilitation therapy; prolonged EN may interfere with these treatments. Therefore, safe administration of EN in a short time is clinically beneficial; conversely, the complication rate associated with rapid administration should be non-inferior to that of conventional administration.

Previous studies have demonstrated the potential for safe and rapid EN administration (9). We hypothesized that rapid EN administration is as safe as conventional administration. Therefore, we planned a multicenter trial to test this hypothesis.

## 2 Methods

### 2.1 Design

**Figure 1:**
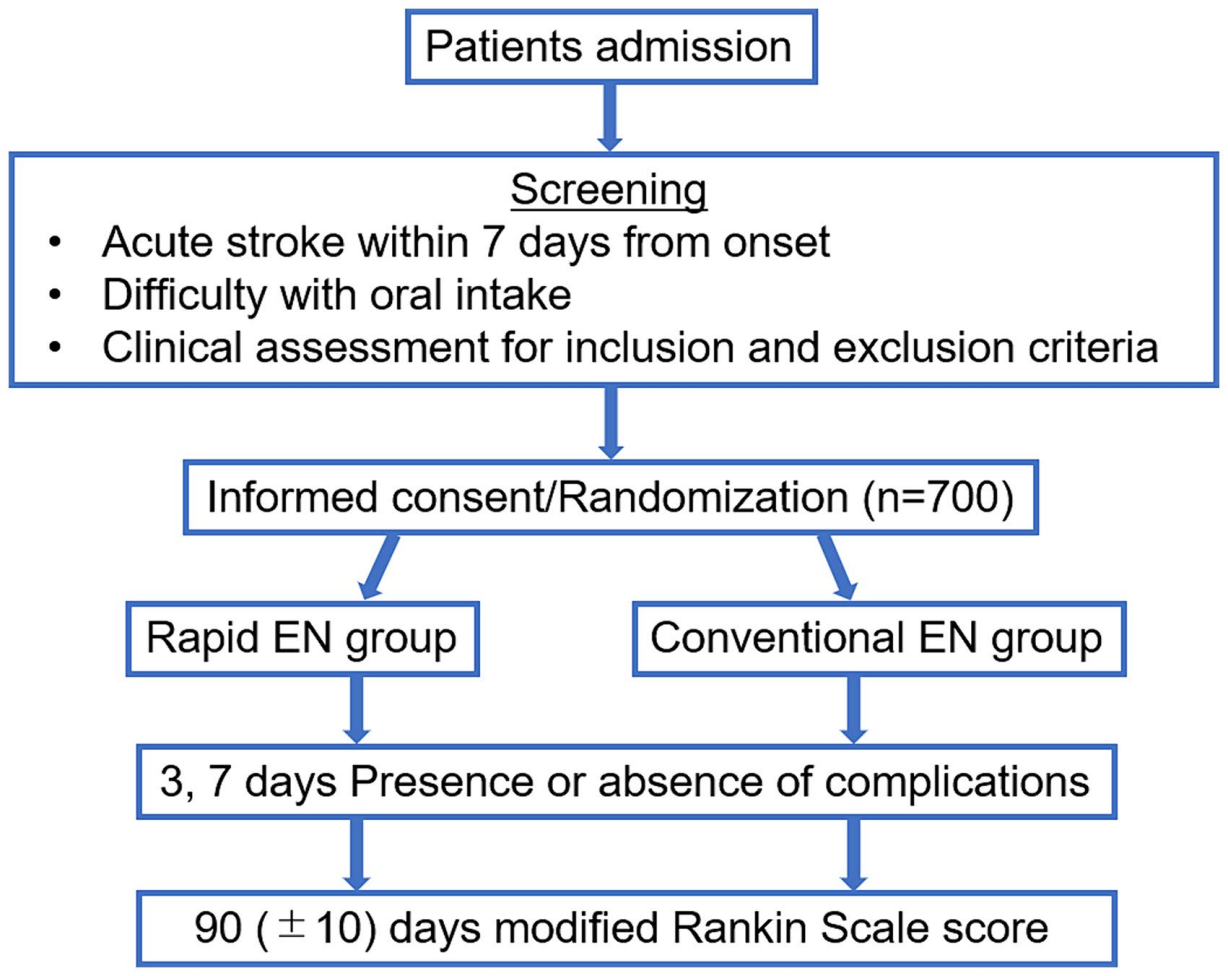
Outline of the study.

This randomized study of rapid EN versus conventional administration in acute stroke patients (Rapid EN Trial) is an investigator-initiated, multicenter, prospective, randomized, open-treatment clinical trial conducted between October 2020 and March 2024. The Institutional Review Board of each hospital approved this trial. All the enrolled patients and their relatives provided written informed consent. This trial was registered as a UMIN Clinical Trial (ID: 000046610). Figure 1 shows a flowchart of the trial design.

### 2.2 Participants

Patients aged ≥20 years who developed stroke within 7 days from onset, had difficulty with oral intake and could raise their head more than 30 degrees represented the target population in the Rapid EN trial. Patients who had difficulty with oral intake were defined as having severely altered consciousness (JCS 10–300), or modified water swallowing test (MWST) (10) <4. Patients with obstructive gastrointestinal tract disease unfit for enteral feeding were excluded. Table 1 shows the inclusion and exclusion criteria.

**Table 1:** Inclusion and exclusion criteria.

Inclusion criteria
∙Age ≥ 18 years at the time of giving informed consent. ∙Clinical diagnosis of acute ischemic stroke within 7 days from onset. ∙Patients who can be randomized within 72 h from admission. ∙Patients who have difficulty with oral intake are defined as severely altered consciousness (JCS 10–300) or modified water swallowing test <4. ∙Patients who can raise their head more than 30 degrees. ∙Written informed consent by the patient or next of kin.
Exclusion criteria
∙Obstructive diseases of the gastrointestinal tract. ∙Pregnancy or possibility of pregnancy. ∙patients with an expected life expectancy of 90 days or less. ∙Patients who are deemed by the investigator or sub-investigator to be unsuitable for participation in this study.

JCS, Japan Coma Scale.

### 2.3 Randomization

Eligible patients are randomized into a 1:1 ratio using a web-based data management system to undergo either rapid administration (rapid EN group) or conventional administration (conventional EN group) of EN within 72 h of admission. Using a minimization algorithm, we balanced the number of patients in the two treatment groups at each hospital.

### 2.4 Treatment or intervention

All the patients received EN using dobhoff tube, NG tube or PEG tube. The enteral feeding dose was 100 mL for the first 3 days and was determined by each institution for each patient for 4–7 days. EN was administered at a rate of 100 mL in 5 min for 7 days in patients randomized to the Rapid EN group and 100 mL in more than 30 min in patients randomized to the Conventional EN group. The type of enteral nutritional supplement is unspecified and freely chosen by each patient.

### 2.5 Clinical assessments

Performances at baseline, three complications of enteral nutrition, including vomiting, diarrhea, and pneumonia, for 3–7 days from the start of EN, location of stroke, the length of hospital stay and clinical outcomes will be assessed by a third independent observer blinded to the group assignment. Vomiting was defined as one or more episodes associated with EN initiation. Diarrhea was defined as a score of 6 or 7 on the Bristol Stool Scale (11), persisting for more than 2 days. Pneumonia was defined as physician-observed pneumonia based on imaging and clinical status. Clinical outcomes were assessed using the modified Rankin scale (mRS) score (range, 0 [no symptoms] to 6 [death]) (12); favorable clinical outcomes were defined as an mRS score of 0–2 at 90 days after stroke onset.

### 2.6 Primary outcome

The primary outcome is the incidence of one or more complications (vomiting, diarrhea, or pneumonia) within 7 days of EN initiation for comparing non-inferiority in the rapid EN and conventional EN groups.

### 2.7 Secondary outcomes

Secondary outcomes were total time spent on EN, incidence of vomiting, diarrhea, and pneumonia within 3 and 7 days of EN, incidence of one or more of the three complications in patients with ischemic or hemorrhagic stroke, and rate of favorable clinical outcome. The efficacy and safety endpoints are presented in Table 2.

**Table 2:** Primary and secondary outcomes.

Primary outcome
∙Incidence of vomiting or diarrhea or pneumonia within 7 days of initiation of enteral nutrition (non-inferiority).
Secondary outcomes
∙Total time spent on enteral nutrition within 7 days of initiation of enteral nutrition (hours).
∙Incidence of vomiting within 7 days of initiation of enteral nutrition.
∙Incidence of diarrhea within 7 days of initiation of enteral nutrition.
∙Incidence of pneumonia within 7 days of initiation of enteral nutrition.
∙Incidence of vomiting or diarrhea or pneumonia within 3 days of initiation of enteral nutrition (non-inferiority).
∙Incidence of vomiting within 3 days of initiation of enteral nutrition.
∙Incidence of diarrhea within 3 days of initiation of enteral nutrition.
∙Incidence of pneumonia within 3 days of initiation of enteral nutrition.
∙Incidence of vomiting or diarrhea or pneumonia within 7 days of initiation of enteral nutrition in ischemic stroke patients (non-inferiority).
∙Incidence of vomiting or diarrhea or pneumonia within 7 days of initiation of enteral nutrition in intracerebral hemorrhage and subarachnoid hemorrhage patients (non-inferiority).
∙Favorable outcome defined as mRS score 0–2 at 90 days after stroke onset.

mRS, modified Rankin Scale.

### 2.8 Sample size estimates

A previous observational study (9) showed that the proportion of patients with one or more complications, defined as vomiting, diarrhea, or pneumonia, was 22/45 (48.9%) in the rapid EN group and 14/26 (53.8%) in the conventional EN group. Based on previous reports, the non-inferiority margin was defined as a minimum clinically important difference (MCID) of 5% in the outcome (13). According to those results, we estimated that 622 patients would need to be enrolled to detect non-inferiority of the rapid EN group compared with the conventional EN group, based on a 1-sided α level of 0.025 and a power of 0.80. In total, 700 patients (350 per intervention and control group) were recruited, accounting for possible treatment failures, protocol violations, and dropouts.

### 2.9 Data monitoring body

Data is monitored centrally by members of the central coordinating center. Members occasionally visit collaborating hospitals to review source materials according to an order from the steering committee. Responsible authorities, related ethics committees, and directors of collaborating hospitals have the right to review the source material if necessary.

### 2.10 Data and safety monitoring board

An independent data and safety monitoring board (DSMB) oversees the conduct of the trial. Any unexpected event is immediately reported to the DSMB. All safety endpoints are analyzed after the inclusion of 100 and 400 patients. In any case of concern regarding the safety of the participants, the steering committee recommend continuing, stopping, or modifying the trial.

### 2.11 Statistical analyses

An intention-to-treat (ITT) analysis will be applied according to the consolidated standards of Reporting Trials (14). As needed, an analysis will be performed on the per-protocol set as a sensitivity analysis of the ITT results. Patient demographic data will be analyzed descriptively. Categorical variables will be assessed using the chi-square test or Fisher’s exact test. In contrast, continuous variables will be assessed using Student’s *t*-test or Wilcoxon rank-sum test, as appropriate. The primary outcome is the proportion of patients with one or more complications at 7 days between the rapid EN and conventional EN groups, as analyzed using univariate logistic regression analysis. Multivariate logistic regression analysis adjusted for potential confounders was performed as a sensitivity analysis. The primary outcome’s odds ratio (OR) will be calculated with the corresponding 95% confidence interval (CI). This clinical trial aimed to demonstrate the non-inferiority of Rapid EN over Conventional EN. If noninferiority is statistically demonstrated for the primary endpoint and the incidence in the rapid EN group is lower than that in the conventional EN group, superiority tests will be implemented. All statistical tests are one-sided, and *p* < 0.025 are considered statistically significant.

### 2.12 Study organization and funding

The Rapid EN trial is organized by a central coordinating center located at the Nippon Medical School and is conducted in approximately 16 centers in Japan. The Rapid EN trial was funded by JSPS Kakenhi Grants (Number 20K19651 to Kentaro Suzuki).

## 3 Discussion and conclusion

Enteral nutrition is widely used worldwide as a nutritional therapy for patients who are unable to consume food orally. Recently, it has been recommended that nutritional therapy be initiated as early as possible in stroke patients (15). However, in actual clinical situations, administering tube feedings three times a day is not only burdensome for medical staff but can also cause psychological stress in acute stroke patients. Therefore, it is reasonable to reduce administration time. Although no previous reports have focused on the speed of administration, most medical staff believe that the rapid administration of enteral nutrition increases the risk of complications such as vomiting, diarrhea, and pneumonia. Therefore, it is necessary to confirm the safety of rapid administration. We believe this study is novel and useful in clinical settings.

An adequate swallowing assessment is important for identifying patients eligible for enteral nutrition. The European Stroke Organization found moderate-quality evidence to recommend dysphagia screening in all stroke patients to prevent poststroke pneumonia (16). Although many swallowing assessment scales exist, the MWST (10) is used in daily practice and included as an eligibility criterion for this study.

This study had several limitations. First, this study allows free choice in the type of enteral nutrition, although this may affect the rate of complications. Differences by type of enteral nutrition will be clarified in a subanalysis. Second, it may be difficult to distinguish vomiting due to brain injury and enteral nutrition. Third, this study cannot examine differences in delivery methods, such as nasal tube versus gastrostomy. Finally, diarrhea can occur secondary to infection which might also be difficult to distinguish from enteral nutrition.

If this trial shows positive results, it will benefit patients and reduce the burden of medical care. Furthermore, this trial could impact healthcare guidelines and costs. We may conduct a meta-analysis of ongoing EN trials for enteral nutrition, including this Rapid EN trial.

## Data availability statement

The datasets presented in this study can be found in online repositories. The names of the repository/repositories and accession number(s) can be found in the article/supplementary material.

## Ethics statement

The studies involving humans were approved by C-2021-031/Nippon Medical School Hospital Drug Trial Review Committee. The studies were conducted in accordance with the local legislation and institutional requirements. The participants provided their written informed consent to participate in this study.

## Author contributions

KS: Writing – original draft, Writing – review & editing. HO: Data curation, Writing – review & editing. RSu: Data curation, Writing – review & editing. SO: Data curation, Writing – review & editing. NK: Data curation, Writing – review & editing. SKa: Data curation, Writing – review & editing. RSe: Data curation, Writing – review & editing. SF: Data curation, Writing – review & editing. KN: Data curation, Writing – review & editing. TT: Data curation, Writing – review & editing. IK: Data curation, Writing – review & editing. HN: Data curation, Writing – review & editing. TK: Data curation, Writing – review & editing. MO: Data curation, Writing – review & editing. SKi: Data curation, Writing – review & editing. SS: Data curation, Writing – review & editing. HK: Data curation, Writing – review & editing. TO: Data curation, Writing – review & editing. KK: Writing – original draft, Writing – review & editing.
